# Hypothalamic over-expression of VGF in the Siberian hamster increases energy expenditure and reduces body weight gain

**DOI:** 10.1371/journal.pone.0172724

**Published:** 2017-02-24

**Authors:** Jo E. Lewis, John M. Brameld, Phil Hill, Cristina Cocco, Barbara Noli, Gian-Luca Ferri, Perry Barrett, Francis J. P. Ebling, Preeti H. Jethwa

**Affiliations:** 1 School of Biosciences, University of Nottingham, Sutton Bonington Campus, Loughborough, United Kingdom; 2 School of Life Sciences, University of Nottingham Medical School, Queen’s Medical Centre, Nottingham, United Kingdom; 3 Department of Biomedical Science, University of Cagliari, 09042 Monserrato, Cagliari, Italy; 4 Rowett Institute for Nutrition and Health, University of Aberdeen, Aberdeen, United Kingdom; University of Minnesota Twin Cities, UNITED STATES

## Abstract

VGF (non-acronymic) was first highlighted to have a role in energy homeostasis through experiments involving dietary manipulation in mice. Fasting increased VGF mRNA in the Arc and levels were subsequently reduced upon refeeding. This anabolic role for VGF was supported by observations in a VGF null (VGF^-/-^) mouse and in the diet-induced and gold-thioglucose obese mice. However, this anabolic role for VGF has not been supported by a number of subsequent studies investigating the physiological effects of VGF-derived peptides. Intracerebroventricular (ICV) infusion of TLQP-21 increased resting energy expenditure and rectal temperature in mice and protected against diet-induced obesity. Similarly, ICV infusion of TLQP-21 into Siberian hamsters significantly reduced body weight, but this was due to a decrease in food intake, with no effect on energy expenditure. Subsequently NERP-2 was shown to increase food intake in rats via the orexin system, suggesting opposing roles for these VGF-derived peptides. Thus to further elucidate the role of hypothalamic VGF in the regulation of energy homeostasis we utilised a recombinant adeno-associated viral vector to over-express VGF in adult male Siberian hamsters, thus avoiding any developmental effects or associated functional compensation. Initially, hypothalamic over-expression of VGF in adult Siberian hamsters produced no effect on metabolic parameters, but by 12 weeks post-infusion hamsters had increased oxygen consumption and a tendency to increased carbon dioxide production; this attenuated body weight gain, reduced interscapular white adipose tissue and resulted in a compensatory increase in food intake. These observed changes in energy expenditure and food intake were associated with an increase in the hypothalamic contents of the VGF-derived peptides AQEE, TLQP and NERP-2. The complex phenotype of the VGF^-/-^ mice is a likely consequence of global ablation of the gene and its derived peptides during development, as well as in the adult.

## Introduction

VGF is a neurotrophin-induced gene that is widely expressed in neuronal and neuroendocrine cells. The VGF gene encodes a 68kDa polypeptide which is cleaved by prohormone convertases (PCs) into multiple smaller peptides and released upon depolarising stimuli [1–4]. Whilst VGF mRNA is expressed in many regions of the nervous system, the highest concentrations of VGF immunoreactivity are found in the ventromedial hypothalamus, in particular the arcuate nucleus (Arc) and paraventricular nucleus (PVN) [5–7].

VGF was first highlighted to have a role in energy homeostasis through experiments involving dietary manipulation in mice. Fasting increased VGF mRNA in the Arc, and levels were subsequently reduced upon refeeding [8]. This anabolic role for VGF was supported by observations in a VGF null (VGF^-/-^) mouse [8]. VGF^-/-^ mice are small, lean, hypermetabolic and hyperactive. They consume more food per gram body weight than wildtype littermate controls, and display increased oxygen consumption and locomotor activity [8]. Interestingly, ablation of the VGF gene blocked the development of obesity in diet- and gold-thioglucose mice and VGF^-/-^ mice crossed with the (A^y/a^) (agouti) mouse, whilst weight gain was attenuated in the *ob/ob* mouse [9, 10].

However, this anabolic role for VGF has not been supported by a number of subsequent studies investigating the physiological effects of VGF-derived peptides. Intracerebroventricular (ICV) infusion of TLQP-21 increased resting energy expenditure and rectal temperature in mice and protected against diet-induced obesity [11]. Similarly, ICV infusion of TLQP-21 into Siberian hamsters significantly reduced body weight, but this was due to a decrease in food intake, with no effect on energy expenditure [12]. Subsequently NERP-2 was shown to increase food intake in rats via the orexin system (body weight data not included) [13], suggesting opposing roles for these VGF-derived peptides. More recently, VGF expression was shown to be dependent upon metabolic state in rats [14], whilst in the Siberian hamster, TLQP immunoreactivity was found to be expressed throughout the hypothalamus (the preoptic area, supraoptic nucleus, suprachiasmatic nucleus and median eminence) in axons and perikarya [15]. A possible explanation for the differences observed between the functional studies utilising the different VGF derived peptides [11, 12] and the genetic studies in the VGF^-/-^ mouse [8] is that global ablation of the gene produces an errant phenotype, possibly due to VGF having pleiotropic roles during development and adult life, as recently reviewed [4].

The aim of this study was therefore to better understand the role of VGF in the regulation of energy homeostasis by utilising a recombinant adeno-associated viral vector (AAV) to over-express VGF in the hypothalamus of adult male Siberian hamsters, thus avoiding any developmental effects or associated functional compensation. We previously demonstrated the feasibility of using the viral 2A sequence in combination with AAV for the long-term over-expression of VGF and fluorescent reporter (eGFP) genes in the hypothalamus of the Siberian hamster [16].

## Methods

### Animals

Male Siberian hamsters (*Phodopus sungorus*) aged 3 months were taken from a colony maintained by the University of Nottingham (Ebling, 1994). Hamsters were housed in individual cages under controlled temperature (21±1°C) and on a reverse photoperiod of 16h light/8h dark (lights off at 11:00h), with *ad-libitum* access to food and water, unless otherwise stated. The diet was standard laboratory chow comprising of 19% extruded protein and 9% fat (Teklad 2019, Harlan, UK). All animal procedures were approved by the University of Nottingham Animal Welfare and Ethical Review Board and were carried out in accordance with the UK Animals (Scientific Procedures) Act 1986 (project licence PPL 40⁄3604).

### Synthesis of construct and viral particles

Synthesis of constructs and viral particles, including *in vitro* and *in vivo* validation, has been described previously [16]. In brief, pAAV-CBA-VGF-2A-eGFP (subsequently called pAAV-VGF-GFP) was constructed by removing the AgRP-IRES-eGFP from the plasmid pAAV-CBA-AgRP-IRES-eGFP-WPRE (a kind gift from Dr Miguel Sena-Esteves (University of Massachusetts, Worcester, USA; [17]) and inserting VGF-2A-eGFP. The plasmid was then sequenced to confirm removal of AgRP-IRES-eGFP and insertion of VGF-2A-eGFP in the correct orientation. Following *in vitro* validation in the SH-SY5Y neuroblastoma cell line, the pAAV-VGF-GFP plasmid was packaged into AAV-2 by Vector BioLabs (PA, USA) and the packaged AAV-GFP control was purchased from Vector Biolabs (PA, USA).

### Infusion of viral constructs

Animal surgical procedures were carried out as previously described [16, 17]. Briefly, animals were placed in a Kopf stereotaxic frame (David Kopf Instruments, NY, USA) with the incisor bar positioned level with the interaural line under general anaesthesia (0.5–2.5% isoflurane). Analgesia was maintained via subcutaneous injection of carprofen (50 mg/kg Rimadyl, Pfizer, Kent, UK). Using the sutures confluence bregma as a landmark, a small hole was drilled on midline and the dura mater was pierced just lateral to the mid-sagittal sinus. A drawn glass capillary microinjector (30-micron tip diameter) was lowered to the correct location. Using a Nanolitre Injection system (WPI, Stevenage, UK) 200nl of the viral vector (AAV-GFP, n = 3 or AAV-VGF-GFP, n = 4) was directed towards the PVN (anteroposterior +0.03, mediolateral ± 0.03, dorsoventral -0.58 (co-ordinates from [18]). Infusions were over two minutes, however the glass microinjector was kept in place for an additional 5 minutes to allow for diffusion and prevention of backflow through the cannula track, and the incision was closed using Michel clips. The surgically-prepared Siberian hamsters were allowed a seven day recovery period, during which they were handled on a daily basis, received analgesia and had access to a palatable diet consisting of soaked Teklab diet. Over-expression of VGF mRNA and eGFP was previously described (16).

### Metabolic gases and feeding behaviour

Multiple respiratory and feeding behaviour parameters were measured using a Comprehensive Lab Animal Monitoring System (CLAMS; Linton Instrumentation, Linton, UK, and Columbus Instruments, Columbus, OH, USA) as described previously [16, 17, 19]. This is an open-circuit calorimeter configured for small rodents, where the rodents were individually housed with food hoppers in the centre of each cage containing chow ground into a rough powder, and dropper-style water bottles. Metabolic parameters measured included oxygen consumption (VO_2_) and carbon dioxide production (VCO_2_), normalised over the estimated lean mass (BW 0.75) due to the change in body composition, such that the energy expenditure (EE) and respiratory exchange ratio (RER) could be calculated as previously described [20]. Feeding behaviour parameters measured included the timing and duration of feeding, individual meal size, and total food intake per unit time. A meal equal to or greater than 0.02g is considered to be a feeding bout. Ambulatory (locomotor) activity was also measured continuously using two sets of infrared beams traversing each cage that measure linear and vertical movement. The system was operated with an air intake of 0.6 L⁄min for each chamber, and an extracted outflow of 0.4 L⁄min. All measurements were taken at an ambient temperature of 21–22°C.

### Effect of over-expression of VGF in the hypothalamus of Siberian hamsters

Three groups of adult male Siberian hamsters were utilised. Group 1 received a bilateral infusion of 200nl of either AAV-GFP (n = 3, 1 x 10^13^ genomic copies/ml, one animal from this group was euthanized due to poor recovery from anaesthesia) as control or AAV-VGF-GFP (n = 4, 7.2 x 10^12^ genomic copies/ml) directed towards the PVN. We have previously shown this to be an effective strategy for infecting the hypothalamus, an area of high VGF expression [17]. After recovery bodyweight and food intake were measured weekly in the home cage. 2 weeks post viral infusion, Group 1 were euthanized by injection of pentobarbital sodium (Euthatal; Rhone Merieux, Harlow, UK) and organs removed, weighed and immediately snap frozen at -80°C. Group 2 received the same vectors as described, were subjected to CLAMS analysis for 48 hours (the first 24 hours were discarded as a period of habituation; the second 24 hours were used for analysis and presented here, a strategy successfully utilised in[16, 17, 19]) at 2 and 12 weeks post viral infusion and subsequently euthanized by injection of pentobarbital sodium and organs removed, weighed and immediately snap frozen at -80°C. Group 3 received the same vectors as described (n = 6 per treatment) and 32 weeks post viral infusion were euthanized by injection of pentobarbital sodium and the brains removed and immediately snap frozen at -80^°^C [16]. eGFP visualization and *in situ* hybridization were performed as previously described [16]. Briefly, antisense transcripts were generated from the pSC-B-AMP/KAN plasmid (containing VGF cDNA) using T7 polymerase (NEB, USA) in the presence of digoxigenin(DIG)/fluorescein-12-uridine-5-triphosphate (a kind gift from Dr Dylan Sweetman, UoN). Riboprobes were purified on a spin column. Slides containing 20μm coronal sections were fixed in 4% PFA/0.1% gluteraldehyde before treatment with proteinase K (10μg/ml). Slides were incubated with hybridization solution containing riboprobe for 6h at 65^°^C. Post-hybrisidation, sections were washed with hybridization solution for 10mins at 65^°^C, followed by two washes with maleic acid buffer containing 0.1% Tween-20 (MABT, pH 7.5). Sections were subsequently blocked in MABT/2% Roche blocking agent for 1h and subsequently incubated overnight with anti-DIG conjugated to alkaline phophatase (1:2000) at 4^°^C. Slides were washed with MABT for 1h followed by an overnight incubation in MABT at 4^°^C. To perform the colour reaction, sections were washed with 1-methyl-5-thiotetrazole (NMTT) containing nitroblue tetrazolium (NBGT) and 5-bromo-3-indocyl-phosphate (BCIP). The colour reaction was stopped by washing the sectionsin 5x TBST (1xTBS, 0.1% Tween 20, 0.2mM sodium azide) overnight at 4^°^C. This process was repeated the following day to intensity the signal and reduce background. Images were captured using a Lecia DMRB microscope (Germany) and OpenLab software (UK). To determine VGF mRNA and GFP expression, slides were scored for the density of signal in the hypothalamic region reflecting hybridization of the VGF probe and GFP signal by an observer who was blind to the treatment: 0 = no hybridization, 1 = a few cells expressing VGF mRNA, 2 = moderate VGF mRNA expression, 3 = abundant VGF mRNA [21].

### Peptide quantification

Quantification of the VGF peptides including, TLQP, AQEE and NERP-2 was carried out via ELISA on whole hypothalamic samples. The antibodies used in each assay were produced against the following peptides: the N-terminal decapeptide of TLQP-21 (rat VGF556-565), AQEE-30 (rat VGF586-595), and the C-terminal nonapeptide of NERP-2 (rat VGF342-350) that contains an amide group at its C-terminus, conjugated with bovine thyroglobulin or keyhole limpet hemocyanin via an additional cysteine at the C-terminus (TLQP, AQEE) or N-terminus (NERP-2). Each antibody has a high affinity for the corresponding VGF peptide, but other cleaved peptides encompassing the sequence could also be recognised, as previously observed with TLQP antiserum which binds to TLQP-21, but also TLQP-62 [15]. The ELISA was carried out as previously described [15]. Briefly, multi-well plates coated with the specific synthetic VGF peptides were incubated with the VGF antisera in parallel with tissue samples and standards (the same synthetic peptides used for immunizations) followed by the relevant biotinylated secondary antibodies (Jackson, West Grove, PA, USA) and the streptavidin-peroxidase conjugate (Biospa, Milan, Italy). Each VGF assay was characterized using various synthetic peptides (see Table 1).

**Table 1:** VGF assay characterization. IC_50_: 50% inhibitory concentration; CV1 and CV2: intra- and inter-assay variation, respectively; h: human; r: rat. ^1^peptide used for plate coating and assay standard. ^2^Des-amidated peptide and ^3^peptide with an additional glycine residue at the C-terminus were used to test for cross reactivity. All of the antisera used for the tissue VGF quantification showed 100% cross-reactivity with the corresponding peptides.

Assay	Peptide	IC_50_ pmol/ml	CV1	CV2	Cross-reactivity
**TLQP**	rVGF_556-564_ (TLQPPASSR)1	1.1	3–5	6–10	100
	rVGF_555-564_				3.5
	rVGF_556-567_ (TLQP-11)				122
	rVGF_556-576_ (TLQP-21)				183
**AQEE**	hVGF_586-595_ (AQEEAEAEER)^1^	3	3–4	10–13	100
**NERP-2**	rVGF_312-350-NH2_^1^	1	3–5	5–8	100
	rVGF_342-350-NH2_				73
	rVGF_342-350_ (des-amide)^2^				<0.001
	rVGF_342-351_ (“G” extended)^3^				<0.001

### Statistical analysis

Descriptive statistics (mean±SEM) were generated using GraphPad Prism (version 6.0, GraphPad Software Inc., San Diego, CA, USA). Body weight, home food intake, data obtained from the CLAMS apparatus and peptide quantification were analysed using two-way repeated measures ANOVA followed by a *post hoc* Bonferroni test. Data on organ weights at the end of the study were analysed using a Student’s unpaired t-test. Scores were analysed by a Kruskal-Wallis test with post-hoc Dunn’s tests for multiple comparisons. Statistical significance was accepted at p<0.05.

## Results

### Over-expression of VGF mRNA

Post mortem analysis revealed high levels of GFP expression in both groups, however, VGF mRNA expression was lowly expressed in the hypothalamus of AAV-GFP animals. Animals treated with AAV-VGF-GFP demonstrated high levels of hypothalamic VGF mRNA which corresponded to the pattern of GFP expression (see S1 Fig).

### Effect of over-expression of VGF on body weight, food intake and ingestive behaviour

**Fig 1:**
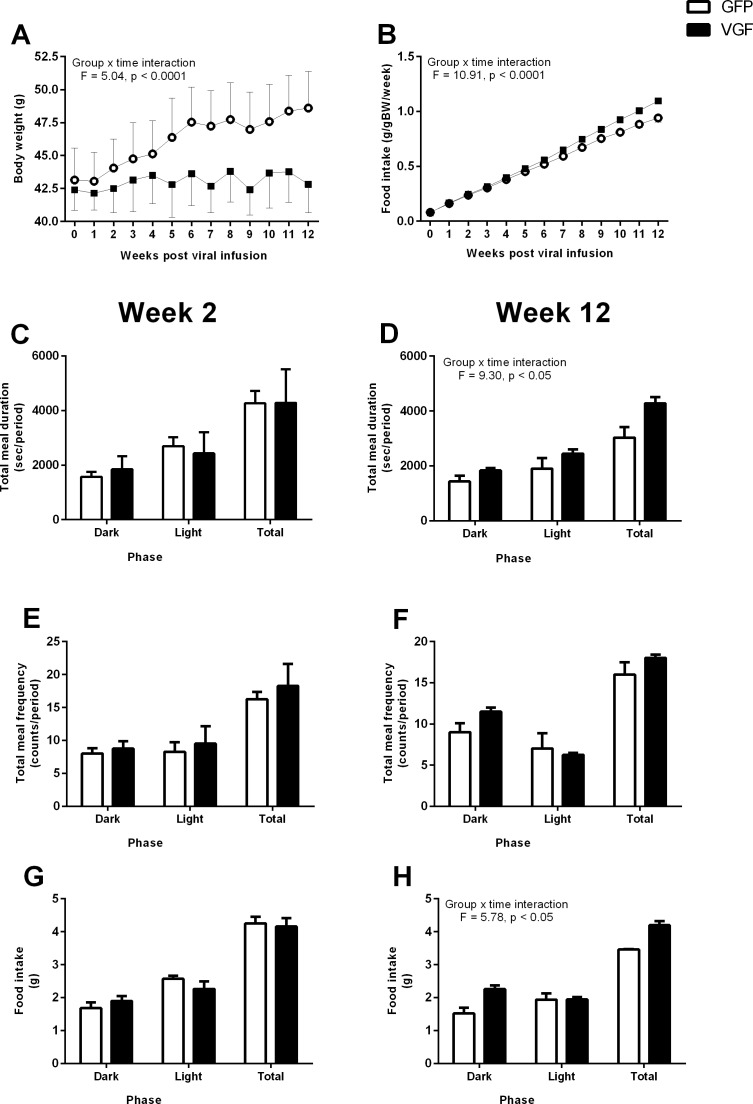
Hypothalamic over-expression of VGF attenuates body weight gain, whilst increasing food intake; a consequence of increased meal duration. Adult male Siberian hamsters received bilateral infusion of either AAV-GFP (control, GFP) or AAV-VGF-GFP (treated, VGF), with bodyweight (in grams) (A) and food intake (grams per gram body weight) (B) determined over the 12 weeks in home cages; while meal duration (C and D) and meal frequency (E and F) were determined over a 24hr period in metabolic cages at 2 (C and E) and 12 weeks (D and F) post-infusion. Values are group mean ±SEM, n = 3–4 per treatment, interaction **** p<0.0001; effect of treatment * p<0.05.

Bilateral infusion of AAV-VGF-GFP into the hypothalamus of Siberian hamsters had no effect on body weight at 2 weeks post infusion compared to AAV-GFP control (Fig 1A), however over the 12 week experimental period an attenuation in the increase in body weight was observed (time vs. treatment interaction F = 5.037, p < 0.001). Siberian hamsters infused with AAV-GFP control increased in bodyweight by an average 12.6% at the end of the 12 week study period whereas those infused with AAV-VGF-GFP only increased by an average of 1.0% (Fig 1A).

Over the 12 week experimental period a significant increase in cumulative food intake was observed in the AAV-VGF-GFP group compared to AAV-GFP control group (time vs. treatment interaction F = 10.91, p <0.0001, Fig 1B). Analysis of the pattern of ingestive behaviour over a 24 hour period at 2 weeks post infusion revealed no effect of treatment on meal duration, frequency and intake (Fig 1C, 1E and 1G, respectively). At 12 weeks post infusion, AAV-VGF-GFP treated animals had a significant increase in meal duration (Fig 1D effect of treatment F = 8.385, p <0.05), but there was no significant change in meal frequency (Fig 1F). Food intake was significantly increased (Fig 1H, effect of treatment F = 5.784, p < 0.05).

### Effect of over-expression of VGF on metabolic parameters

**Fig 2:**
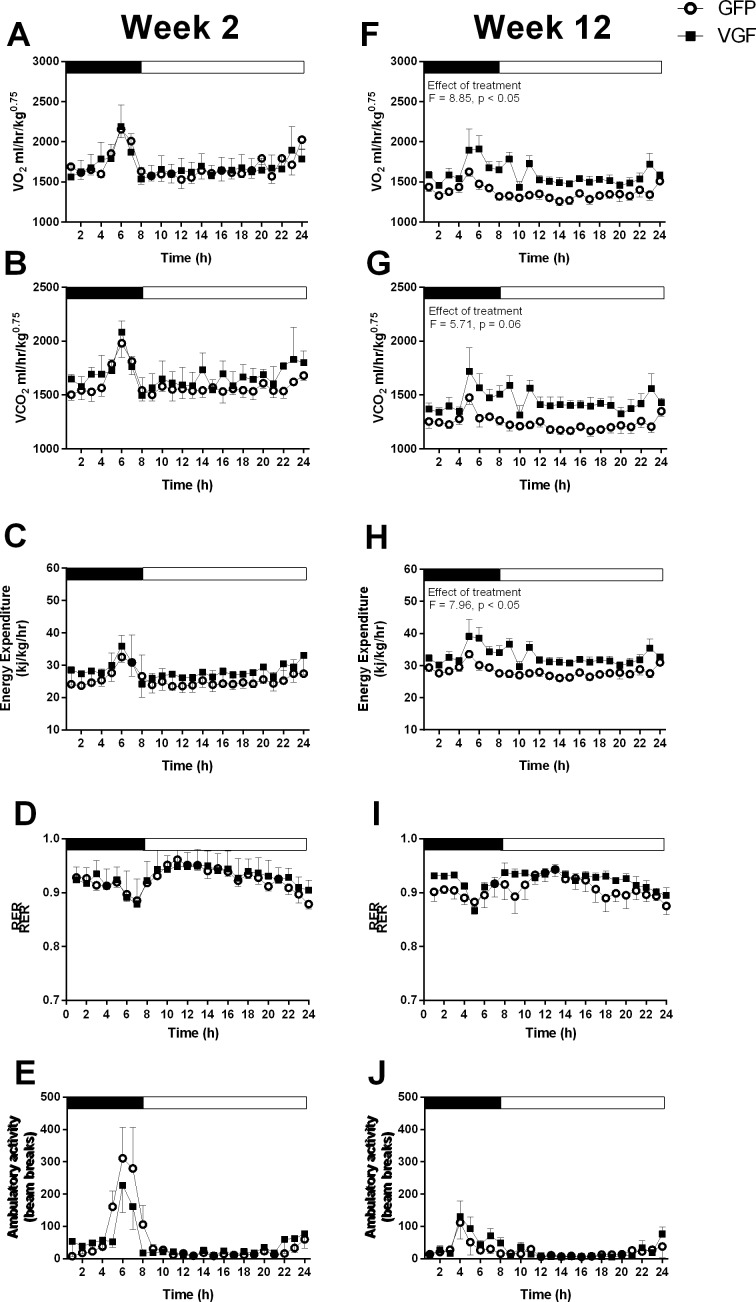
Hypothalamic over-expression of VGF increases oxygen consumption (VO_2_) which is unrelated to ambulatory activity. 24 hour profiles of oxygen consumption (VO_2_) (A and F), carbon dioxide production (VCO_2_) (B and G), energy expenditure (C and H), respiratory exchange ratio (RER) (D and I) and ambulatory (locomotor) activity (E and J) of adult male Siberian hamsters measured in metabolic cages at 2 (A-E) and 12 weeks (F-J) post-infusion with either AAV-GFP (control, GFP) or AAV-VGF-GFP (treated, VGF) viral vectors. Values are group mean ±SEM, n = 3–4 per treatment, effect of treatment * p<0.05.

Analysis of metabolic parameters at 2 weeks post bilateral infusion with AAV-VGF-GFP revealed no effect of treatment at this stage compared to the AAV-GFP control group (Fig 2A–2E). However, by 12 weeks post infusion there was a significant increase in VO_2_ in the AAV-VGF-GFP treated group compared to the AAV-GFP control animals (effect of treatment = 8.854, p< 0.05; Fig 2F), while there was a trend for an increase in VCO_2_ (effect of treatment F = 5.706, p = 0.06; Fig 2G). The effect was primarily in the dark phase, with VO_2_ being 13.4% higher in the AAV-VGF-GFP treated group compared to the AAV-GFP control group. This resulted in a significant increase in energy expenditure in the AAV-VGF-GFP control group (effect of treatment F = 7.968, p <0.05; Fig 2H). No effects on RER (Fig 2I) or ambulatory activity (Fig 2J) were observed.

### Effect of over-expression of VGF on organ weight

There was no effect on organ or tissue weight in Siberian hamsters bilaterally infused with AAV-VGF-GFP (compared to those infused with AAV-GFP as a control) 2 weeks post infusion (Table 2). However, at 12 weeks post infusion, there was an increase in the weight of interscapular brown adipose tissue (p<0.05, Table 2) and a decrease in weight of interscapular white adipose tissue (p< 0.05, Table 2) in the AAV-VGF-GFP group. There were no significant effects on the wet weights of the epididymal white adipose tissue and the liver (Table 2).

**Table 2:** Hypothalamic over-expression of VGF in Siberian hamsters increases BAT weight and reduces interscapular white adipose tissue weight. Mean (± SEM) wet tissue weight (mg per g BW) in Siberian hamsters receiving bilateral AAV-GFP or AAV-VGF-GFP at 2 and 12 weeks post viral infusion. * p < 0.05.

Group	Treatment	LIVER (mg/g BW)	eWAT (mg/g BW)	iBAT (mg/g BW)	iWAT mg/g BW)
Group 1 (week 2)	AAV-GFP	34.9 ± 2.2	25.6 ± 0.7	2.6 ± 0.3	17.4 ± 3.5
	AAV-VGF-GFP	32.9 ± 1.4	27.5 ± 0.6	2.5 ± 0.2	16.3 ± 2.1
Group 2 (week 12)	AAV-GFP	36.1 ± 1.9	23.6 ± 1.6	2.9 ± 0.2	24.4 ± 3.4
	AAV-VGF-GFP	38.0 ± 3.7	22.8 ± 0.5	4.6 ± 0.4*	17.4 ± 3.5*

eWAT = epididymal white adipose tissue; iBAT = intrascapular brown adipose tissue; iWAT = intrascapular white adipose tissue

### Effect of over-expression of VGF peptide levels in the hypothalamus

**Fig 3:**
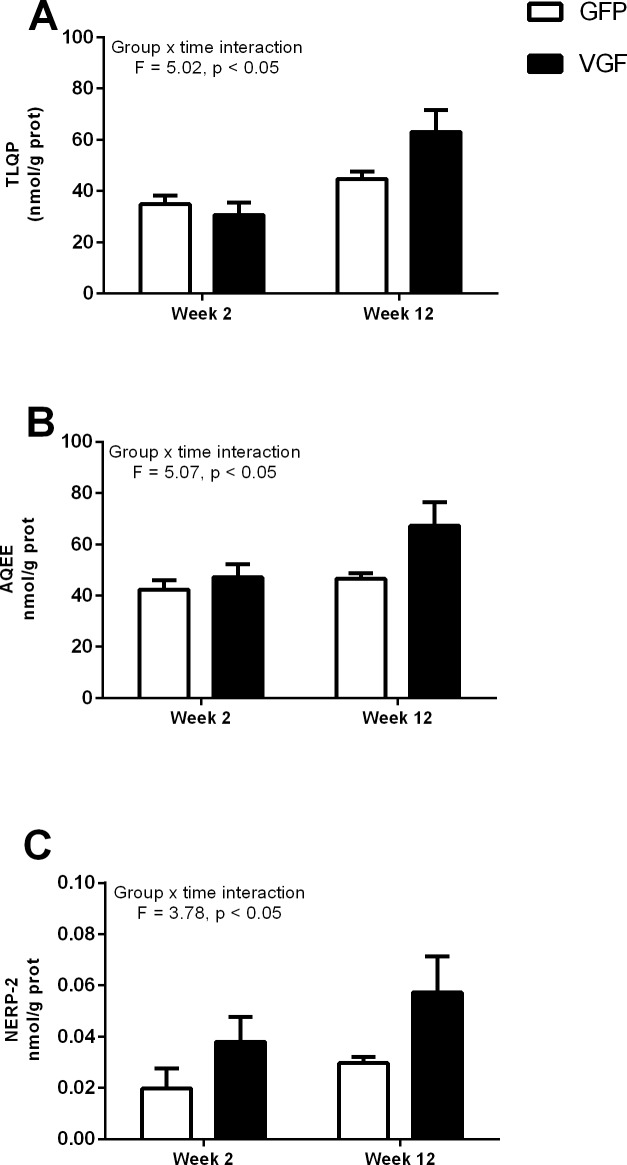
Hypothalamic over-expression of VGF resulted in time-dependent increases in the VGF-derived peptides, TLQP, AQEE and NERP-2, in the hypothalamus of adult male Siberian hamsters. Hypothalamic peptide levels (nmol/g protein) of TLQP (A), AQEE (B) and NERP-2 (C) in adult hamsters bilaterally infused with either AAV-GFP (control, GFP) or AAV-VGF-GFP (treated, VGF) viral vectors at 2 or 12 weeks post infusion. Values are group mean ±SEM, n = 3–4 per treatment, interaction *p<0.05.

Bilateral infusion of AAV-VGF-GFP for two weeks (Group 1) did not result in any changes in the levels of VGF derived peptides TLQP, AQEE and NERP-2 compared to those infused with AAV-GFP, however by 12 weeks an increase in all three peptides was apparent (Fig 3A–3C, time vs. treatment interactions F = 5.02, 5.07 and 3.78 respectively, p<0.05).

## Discussion

Hypothalamic over-expression of VGF in adult Siberian hamsters produced a complex phenotype; at 2 weeks post infusion there was no effect on metabolic parameters. AAV-2 has a relatively slow onset of transcription but has been previously shown to efficiently and stably over-express trans- and reporter-genes [17, 22, 23]. By 12 weeks hamsters had increased oxygen consumption and a tendency to increased carbon dioxide production; though no significant change in RER and therefore no evidence that substrate utilization (carbohydrate vs. fat oxidation) was affected. The increase in oxygen consumption 12 weeks post viral infusion was not due to an increase in locomotor activity, as this was similar in AAV-GFP and AAV-VGF-GFP infused animals. We infer that the significant increase in oxygen consumption, and therefore energy expenditure, 12 weeks post viral infusion, particularly during the dark phase, was the main driver for the attenuated body weight gain in AAV-VGF-GFP infused animals. Indeed, hypothalamic over-expression of VGF reduced interscapular white adipose tissue weights, but increased interscapular brown adipose tissue weights, 12 weeks post infusion, which may potentially account for the increased energy expenditure. Interestingly, externally located WAT depots are relatively spared in response to photoperiod change in the Siberian hamster; epididymal WAT demonstrates proportionally greater decreases [24]. This suggests differential sympathetic neural control in WAT depots in this species. There was a small but significant increase in food consumption measured in the home cage in AAV-VGF-GFP infused animals. The increase in cumulative food intake was apparent 3 weeks post infusion and persisted for the 12 week experimental period; this resulted in significantly increased cumulative food intake (grams per g body weight) in the AAV-VGF-GFP infused group. This reflected increased meal duration and intake, as measured in CLAMS at 12 weeks post viral infusion, since meal frequency was unaffected. There was no effect on these behavioural parameters 2 weeks post infusion. We infer that these increases in food intake were a compensatory mechanism to limit weight loss and maintain energy stores. These observed changes in energy expenditure and food intake were associated with an increase in the hypothalamic contents of the VGF-derived peptides, AQEE, TLQP and NERP-2. These peptides have been shown to be involved regulating energy homeostasis, since ICV administration of TLQP-21 has been shown to reduce food intake in Siberian hamsters [12] and increase energy expenditure in mice [11], while ICV infusion of NERP-2 in rats was shown to increase energy expenditure and food intake via the orexin system [13], suggesting opposing roles for these two distinct VGF derived peptides. These functional studies conducted with VGF derived peptides in multiple species largely support the phenotype produced by hypothalamic over-expression of VGF in the Siberian hamster. Both of these VGF derived peptides (TLQP and NERP-2) were increased 12 weeks post viral infusion with AAV-VGF-GFP and may contribute to the observed increase in energy expenditure. Interestingly, TLQP-62 is the most prominent VGF derived peptide [3], whilst AQEE-30 increases upon high caloric feeding in rats [25] and analysis of hSNP mice (where the C terminal of VGF is deleted) suggests AQEE-30 may have a positive effect on energy homeostasis (as these mice have reduced adiposity) or act as functional antagonists of TLQP-21 under physiological conditions [26]. However, this ablation is once again associated with a robust increase in oxygen consumption, food intake and locomotor activity; a consequence of hyperactivity [26].

Interestingly, hypothalamic knockdown of VGF leads to metabolic disturbances in male mice [27]. Hypothalamic knockdown of VGF, using a Cre-loxP system, resulted in weight gain and decreased body temperature, oxygen consumption, RER and locomotor activity. Whilst food intake was unaffected, glucose tolerance was impaired. These effects resulted in increased adiposity and reduced UCP1 protein in BAT, a phenotype that is mostly opposite (and therefore in agreement with) the phenotype described here for hypothalamic VGF over-expression and largely consistent with the proposed role of TLQP-21 in adult mice [11].

Interestingly, these effects of VGF over-expression are analogous to studies of cocaine amphetamine regulated transcript (CART), which has orexigenic and anorectic effects dependent upon its site of hypothalamic ICV infusion [28–30]. Furthermore, the phenotype produced by the hypothalamic over-expression of VGF in the Siberian hamster is very similar to transgenic mice that over-express orexin, with orexin having been shown to mediate the effects of NERP-2 in rats [13, 31]. Interestingly, these orexin transgenic mice are resistant to diet-induced obesity as a result of increased energy expenditure despite significantly increased daily food intake [31].

It is also of note that VGF gene expression is photoperiodically regulated, with short day length (SD) which leads to reduced adiposity, associated with a decrease in expression of VGF in the Arc but dramatically higher expression in the dorsomedial posterior Arc of Siberian hamsters [21, 32]. Given the nature of the phenotype produced by hypothalamic over-expression of VGF in Siberian hamsters in long day length (LD) described here, there is a need to determine peptide levels in specific hypothalamic nuclei to further elucidate their role in seasonal adaptation.

The findings of the studies presented here are largely in agreement with those of Bartolomucci et al. [11] and Jethwa et al. [12], which both utilised the VGF derived peptide TLQP-21, as well as the hypothalamic knockdown studies conducted by Foglesong et al. [27]. However, they are in contrast with the VGF^-/-^ and hSNP mice, which are lean, hypermetabolic and hyperactive [8, 26]. The complex phenotype of the VGF^-/-^ mice [8] is a likely consequence of global ablation of the gene and its derived peptides during development, as well as in the adult. Hahm et al. [8] postulated that an increase in VGF expression in the hypothalamus of mice would increase food intake and body weight, while energy expenditure would decrease, resulting in an obese phenotype. Indeed germline over-expression of VGF in mice modestly increased body weight and food intake, whilst reducing locomotor activity [26]. The current study demonstrates that over-expression of VGF in the hypothalamus of the Siberian hamster actually results in the opposite phenotype, increasing energy expenditure and reducing body weight gain, despite increasing food intake, highlighting the complexity of VGF and its derived peptides during development and adulthood.

## Supporting information

S1 Fig Hypothalamic over-expression of VGF mRNA corresponds to GFP expression in AAV-VGF-GFP treated animals. Hypothalamic VGF mRNA is increased in AAV-VGF-GFP treated animals and is limited to a few cells in the AAV-GFP group (if detected) despite high levels of GFP expression. Values are group mean ±SEM, n = 6 per treatment.
(PDF)
