# Correction: Hypothalamic over-expression of VGF in the Siberian hamster increases energy expenditure and reduces body weight gain

**DOI:** 10.1371/journal.pone.0182594

**Published:** 2017-07-28

**Authors:** Jo E. Lewis, John M. Brameld, Phil Hill, Cristina Cocco, Barbara Noli, Gian-Luca Ferri, Perry Barrett, Francis J. P. Ebling, Preeti H. Jethwa

The following information is missing from the Funding section: This work was supported by the Biotechnology and Biological Sciences Research Council [grant numbers RS3522, F53511 RTSG].
