# Supplementary figures and images for: Hypothalamic over-expression of VGF in the Siberian hamster increases energy expenditure and reduces body weight gain
Source: PLoS One. 2017 Feb 24;12(2):e0172724. doi: 10.1371/journal.pone.0172724 (PMC5325529; doi:10.1371/journal.pone.0172724)

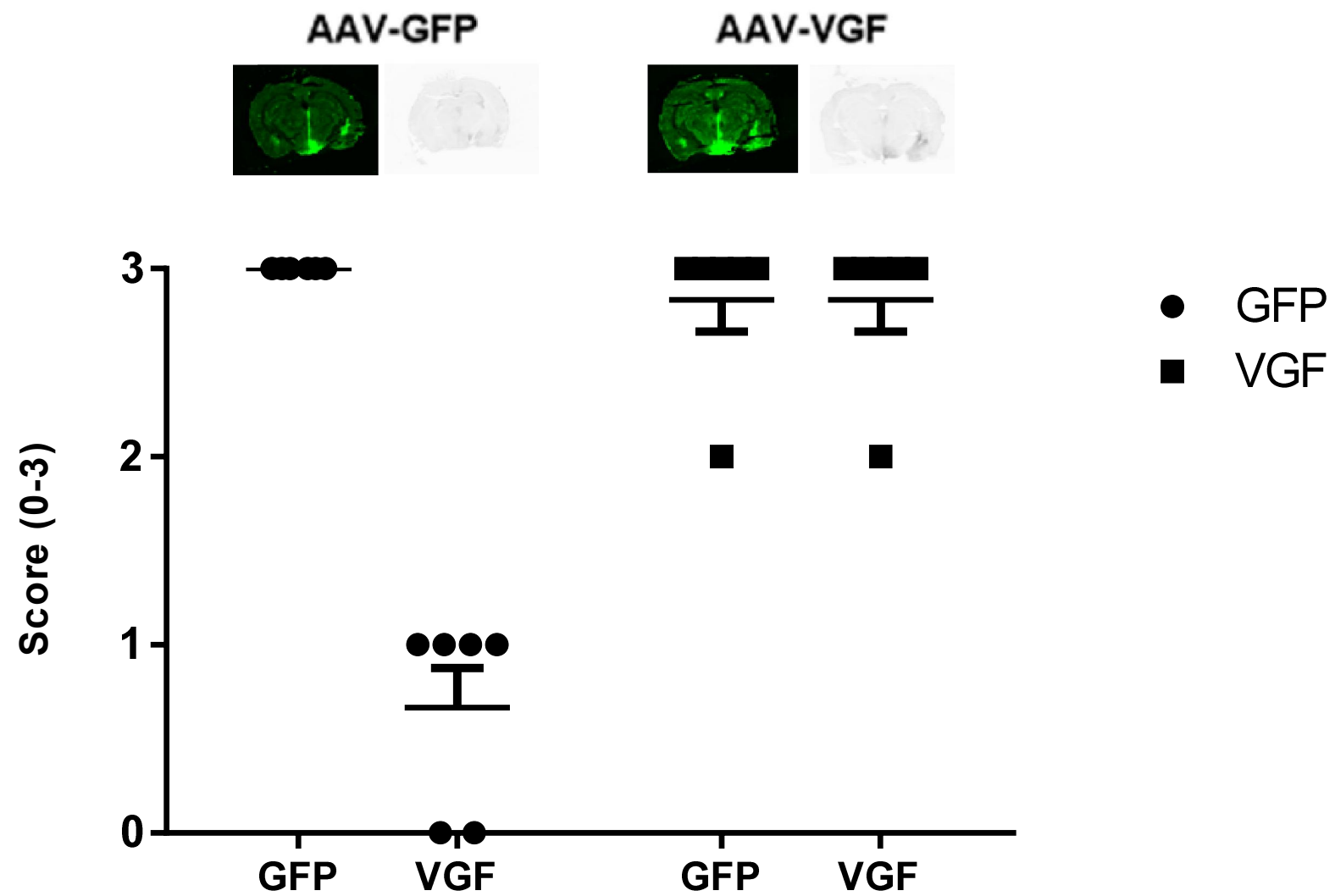

Supplement: S1 Fig — Hypothalamic VGF mRNA is increased in AAV-VGF-GFP treated animals and is limited to a few cells in the AAV-GFP group (if detected) despite high levels of GFP expression. Values are group mean ±SEM, n = 6 per treatment. (PDF) [file pone.0172724.s001.pdf]
